# Applications of artificial intelligence in interventional oncology: An up-to-date review of the literature

**DOI:** 10.1007/s11604-024-01668-3

**Published:** 2024-10-02

**Authors:** Yusuke Matsui, Daiju Ueda, Shohei Fujita, Yasutaka Fushimi, Takahiro Tsuboyama, Koji Kamagata, Rintaro Ito, Masahiro Yanagawa, Akira Yamada, Mariko Kawamura, Takeshi Nakaura, Noriyuki Fujima, Taiki Nozaki, Fuminari Tatsugami, Tomoyuki Fujioka, Kenji Hirata, Shinji Naganawa

**Affiliations:** 1 Department of Radiology, Faculty of Medicine, Dentistry and Pharmaceutical Sciences, Okayama University, 2-5-1 Shikata-Cho, Kita-Ku, Okayama 700-8558 Japan; 2 Department of Artificial Intelligence, Graduate School of Medicine, Osaka Metropolitan University, Abeno-Ku, Osaka Japan; 3 Department of Radiology, Graduate School of Medicine and Faculty of Medicine, The University of Tokyo, Bunkyo-Ku, Tokyo Japan; 4 Department of Diagnostic Imaging and Nuclear Medicine, Kyoto University Graduate School of Medicine, Sakyoku, Kyoto Japan; 5 Department of Radiology, Kobe University Graduate School of Medicine, Chuo-Ku, Kobe Japan; 6 Department of Radiology, Juntendo University Graduate School of Medicine, Bunkyo-Ku, Tokyo Japan; 7 Department of Radiology, Nagoya University Graduate School of Medicine, Showa-Ku, Nagoya Japan; 8 Department of Radiology, Osaka University Graduate School of Medicine, Suita-City, Osaka Japan; 9 Medical Data Science Course, Shinshu University School of Medicine, Matsumoto, Nagano Japan; 10 Department of Diagnostic Radiology, Kumamoto University Graduate School of Medicine, Chuo-Ku, Kumamoto Japan; 11 Department of Diagnostic and Interventional Radiology, Hokkaido University Hospital, Kita-Ku, Sapporo Japan; 12 Department of Radiology, Keio University School of Medicine, Shinjuku-Ku, Tokyo Japan; 13 Department of Diagnostic Radiology, Hiroshima University, Minami-Ku, Hiroshima Japan; 14 Department of Diagnostic Radiology, Tokyo Medical and Dental University, Bunkyo-Ku, Tokyo Japan; 15 Department of Diagnostic Imaging, Graduate School of Medicine, Hokkaido University, Kita-Ku, Sapporo Japan

**Keywords:** Artificial intelligence, Machine learning, Interventional radiology, Oncology, Embolization, Ablation

## Abstract

Interventional oncology provides image-guided therapies, including transarterial tumor embolization and percutaneous tumor ablation, for malignant tumors in a minimally invasive manner. As in other medical fields, the application of artificial intelligence (AI) in interventional oncology has garnered significant attention. This narrative review describes the current state of AI applications in interventional oncology based on recent literature. A literature search revealed a rapid increase in the number of studies relevant to this topic recently. Investigators have attempted to use AI for various tasks, including automatic segmentation of organs, tumors, and treatment areas; treatment simulation; improvement of intraprocedural image quality; prediction of treatment outcomes; and detection of post-treatment recurrence. Among these, the AI-based prediction of treatment outcomes has been the most studied. Various deep and conventional machine learning algorithms have been proposed for these tasks. Radiomics has often been incorporated into prediction and detection models. Current literature suggests that AI is potentially useful in various aspects of interventional oncology, from treatment planning to post-treatment follow-up. However, most AI-based methods discussed in this review are still at the research stage, and few have been implemented in clinical practice. To achieve widespread adoption of AI technologies in interventional oncology procedures, further research on their reliability and clinical utility is necessary. Nevertheless, considering the rapid research progress in this field, various AI technologies will be integrated into interventional oncology practices in the near future.

## Introduction

The applications of artificial intelligence (AI) in medicine are rapidly advancing and becoming widespread. The field of radiology is considered particularly well suited for incorporating AI technologies because of the high image-processing capabilities of current AI models. AI has been applied to various aspects of diagnostic radiology and nuclear medicine across various imaging modalities and target organs [1–6]. Studies have shown that AI can be useful for lesion detection [7–10], differential diagnosis [11–15], and image quality improvement [16–21]. Furthermore, the application of AI has been increasingly reported in the field of radiation therapy, where it is used to support various tasks during treatment, including preparation, delivery, and evaluation [22].

The application of AI is being explored also in interventional radiology, a specialty that offers image-guided, minimally invasive therapies [23, 24]. The literature has shown the potential of AI-based tools for intraprocedural support and pre/post-procedural assessment in various interventional radiology fields, including neurointervention, aortic and peripheral vascular intervention, and coronary intervention [25, 26]. Interventional oncology, a subspecialty of interventional radiology, offers image-guided interventions for malignant tumors, with AI-based technologies expected to play a significant role. The key treatments in interventional oncology include transarterial tumor embolization and percutaneous tumor ablation for lesions in various organs [27–32]. In these treatments, imaging is crucial at every stage, from deciding on treatment indications to planning, performing procedures, and post-treatment follow-up. Consequently, interventional oncology may potentially benefit from rapid advancements in AI-based image-processing technologies, leading to significant interest and an increase in relevant studies. This review outlines the current research on AI applications in interventional oncology based on the latest literature.

## Overview of literature

A literature search was conducted for this narrative review in June 2024, using PubMed with the following terms: “artificial intelligence,” “machine learning,” “deep learning,” or “neural network,” and “interventional oncology,” “tumor ablation,” “radiofrequency ablation,” “microwave ablation,” “cryoablation,” “embolization,” “chemoembolization,” or “radioembolization.” Notably, 90% (332/371) of the articles identified in the search were published in 2020 or later, indicating a recent rapid increase in research on this topic. We screened these articles and extracted relevant studies for review, primarily focusing on those associated with the clinical application of AI in interventional oncology and excluding those that solely employed animal experimental data or focused on AI methodologies. Additionally, we reviewed several relevant articles found through a manual search of the citations in the reviewed articles or through personal communication.

**Fig. 1:**
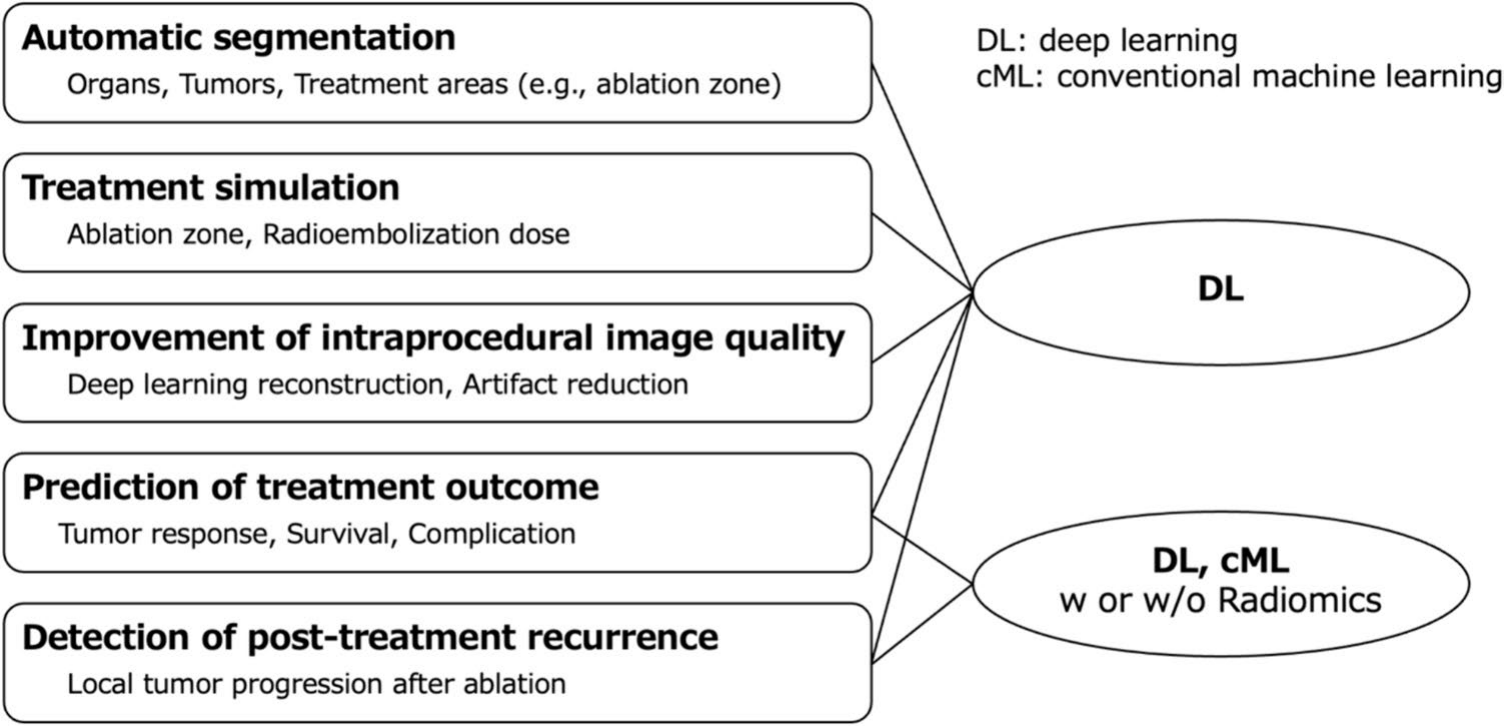
Application of artificial intelligence in interventional oncology. Deep learning could be utilized for the various tasks mentioned in this review. For outcome prediction and recurrence detection, deep learning or conventional machine learning, with or without radiomics, could be employed

In the reviewed studies, the application of AI has been attempted in various tasks, including automatic segmentation of organs, tumors, and treatment areas; treatment simulation; improvement of intraprocedural image quality; prediction of treatment outcomes; and detection of post-treatment recurrence (Fig. 1). Among these, the prediction of treatment outcomes has been the most studied. From a technical perspective, the investigators have used deep and conventional machine learning, sometimes comparing these approaches. Here, “conventional machine learning” refers to the machine learning algorithms that have been widely used since the time before the rise of deep learning, including logistic regression, support vector machine, and random forest [33, 34]. These algorithms make decisions using specific functions based on manually selected and engineered features. Deep learning, a subset of machine learning, is based on neural networks, particularly those with multiple layers (33). Deep learning models automatically extract and learn features from data to make decisions with minimal human intervention. In addition, investigators often incorporate machine learning techniques into the radiomics process. Radiomics involves extracting numerous quantitative features that are invisible to the human eye from medical images, which are then analyzed and used to construct models for disease diagnosis, treatment evaluation, and prognostication [35]. In the following sections, we describe how these AI technologies can be applied to interventional oncology based on the literature.

## Automatic segmentation of organs, tumors, and treatment areas

AI can potentially enable automatic segmentation of organs, tumors, and treatment areas in interventional procedures, which may contribute to precise tumor targeting, objective evaluation of treatment areas, and potentially lead to a streamlined procedural workflow. Accordingly, some investigators developed AI-based automatic segmentation algorithms using data from patients undergoing image-guided tumor ablation. He et al. investigated a deep learning-based method for segmenting the liver, tumor, and ablation zone using computed tomography (CT) before and after ablation therapy [36]. They trained a residual attention U-net (a U-shaped fully convolutional neural network [CNN]) model using the public dataset of the Liver Tumor Segmentation Challenge (LiTS) [37] and their local dataset of 48 patients who underwent radiofrequency ablation (RFA) or microwave ablation (MWA) for liver tumors. In the test set, their model achieved dice similarity coefficients (DSC) of 0.96, 0.64, and 0.83 for liver, tumor, and ablation zone segmentation in the arterial phase images, where a DSC score closer to 1 indicates a higher overlap between the predicted and true segments. Fang et al. also developed a deep learning-based liver segmentation algorithm using the LiTS dataset and demonstrated that the segmentation method was useful for surface-based image fusion of intraprocedural CT and preprocedural magnetic resonance imaging (MRI), contrast-enhanced CT, or positron emission tomography (PET)/CT images to facilitate tumor targeting [38]. Similar automatic segmentation methods for lung-tumor ablation have been investigated. Mahmoodian et al. developed U-Net-based segmentation models using CT data obtained during CT-guided lung MWA in 50 patients [39]. In their best model, the intersection over union (IoU) values for lung, ablated tissue, and tumor segmentation were 0.98, 0.77, and 0.54, respectively. Here, the IoU was calculated as the area of overlap between the predicted and true segmentations divided by the area of their union, and an IoU value closer to 1 indicates a higher degree of overlap between the segments [40, 41]. Zhou et al. evaluated a U-net algorithm for lung nodule segmentation on preprocedural CT in 55 patients who underwent RFA and obtained DSC and IoU values of 0.88 and 0.88, respectively [42].

Deep learning-based segmentation methods may also be useful for transarterial treatments. Malpani et al. developed a U-net model for the segmentation of lipiodol deposition on cone-beam CT after transarterial chemoembolization (TACE) of liver tumors and compared it to a thresholding method (a method that delineates the lipiodol deposition area based on CT value thresholds) [43]. The U-net model performed better than the thresholding method (DSC; 0.65 vs. 0.45, *p* < 0.001) when segmentation by an experienced radiologist was used as the ground truth. The difference between the predicted and actual center of mass was smaller with the U-net model than with the thresholding method (15.31 mm vs. 31.34 mm, *p* < 0.001), indicating the higher accuracy of the U-net model. Chaichana et al. developed a CNN-based model for the automated segmentation of the lung, liver, and tumors on technetium-99 macroaggregated albumin (^99m^Tc-MAA) single-photon emission CT (SPECT)/CT images for planning yttrium-90 (^90^Y) radioembolization of liver tumors [44]. The authors trained the model using images from 56 patients with hepatocellular carcinoma (HCC), which showed DSC of 0.98, 0.91, and 0.85 in the segmentation of lungs, liver, and tumors, respectively, in the test sets. In ^90^Y radioembolization, accurate segmentation of targets and organs at risk on pretreatment ^99m^Tc-MAA SPECT/CT is pivotal for precisely predicting microsphere distribution and dose estimation. As segmentation is usually performed manually and is time-consuming, AI-based methods could be of great help in this task.

## Treatment simulation

A robust simulation of technical results is crucial for optimizing treatment methods when planning interventional oncology procedures. Some investigators are exploring deep learning-based simulation for image-guided tumor ablation and transarterial radioembolization.

### Simulation of ablation zone in ablative therapies

Covering the target tumor with an adequate margin in the ablation zone is necessary to ensure local control during image-guided tumor ablation. The position of the ablation probe is carefully planned before the procedure to achieve an appropriate ablation zone, usually using the vendor’s chart for the expected ablation-zone dimensions. However, these vendor data, based on ex vivo animal experiments, often differ significantly from actual patient results because of various factors, such as the local anatomy of each case. For instance, nearby blood vessels can affect heat-based ablation by causing a heat-sink effect [45] or cryoablation by causing a cold-sink effect [46], resulting in narrower ablation zones. Therefore, to accurately predict the ablation zone before the procedure, some investigators have turned to deep learning methods. Keshavamurthy et al. introduced a deep learning model that predicts the ablation zones of lung MWA based on preprocedural CT images, ablation power and time, and applicator position [47]. Data from 52 ablation procedures performed on 40 patients were used and the ablation zones manually segmented on post-treatment images by an experienced radiologist served as the ground truth. Their model outperformed the vendor model (expected ablation zones based on the vendor data) in predicting the ablation zone in the test set (DSC: 0.62 vs. 0.56). Notably, their model could simulate the deformation of the ablation zone caused by the heat-sink effect of blood vessels and the marginal shape along organ boundaries. Moreira et al. reported a deep learning model based on a 3D U-net to predict the ablation zone in cryoablation (iceball) from the position of cryoprobes [48]. The model was trained using the intraprocedural MRI of 38 patients undergoing cryoablation for prostate cancer and predicted the extent of the iceball more accurately than that by the vendor model (DSC: 0.79 vs. 0.72, *p* < 0.001). There was no significant difference between the iceball volume predicted by the model and the ground truth, whereas the volume predicted by the vendor model was significantly smaller than that of the ground truth.

### Simulation of absorbed dose in radioembolization

When calculating the expected absorbed doses in ^90^Y radioembolization therapy, the dose estimation model assumes that the biodistribution of ^90^Y microspheres in the areas of interest is uniform. However, the estimated absorbed dose based on pretreatment ^99m^Tc-MAA SPECT/CT often differs significantly from that calculated based on the actual biodistribution of ^90^Y microspheres confirmed by post-treatment PET/CT or SPECT/CT [49]. Inaccurate absorbed-dose estimation may cause erroneous predictions of treatment response, highlighting the need for more accurate pretreatment dose estimation methods. To address this, Plachouris et al. developed a deep learning model that could generate predicted post-treatment ^90^Y PET/CT images based on pretreatment ^99m^Tc-MAA SPECT/CT data to simulate ^90^Y microspheres biodistribution [50]. Their model, a conditional generative adversarial network (GAN) designed for image-to-image translation, was trained using data from 19 patients undergoing radioembolization for primary or metastatic liver tumors, and its performance was evaluated by comparing image-based dosimetry between the predicted and actual PET-CT images. The difference between the mean absorbed dose calculated on the predicted PET-CT and that on the actual PET-CT was 7.98 ± 31.39 Gy and 0.03 ± 0.25 Gy for the tumor and non-tumoral liver, respectively, suggesting that their deep learning method provided more accurate dose prediction than that by existing methods.

## Improvement of intraprocedural image quality

**Fig. 2:**
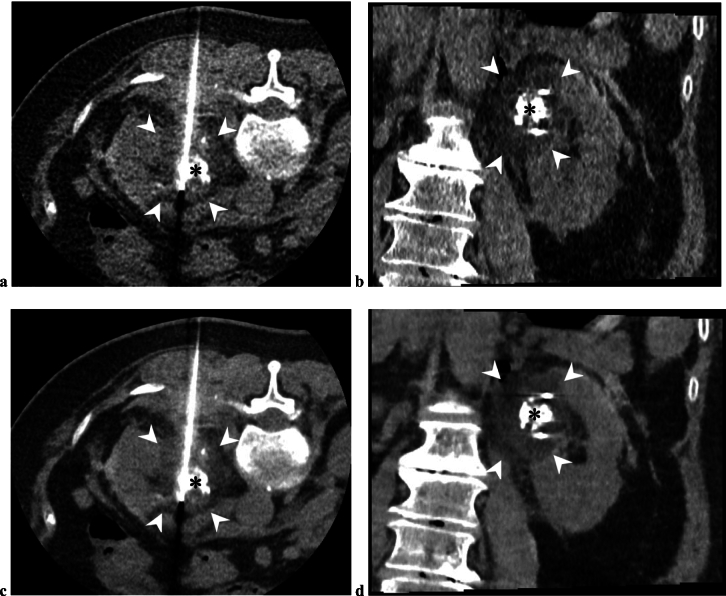
CT images immediately after freezing in a cryoablation procedure for a left renal cell carcinoma, with the patient in the prone position (**a**, **c**: axial section; **b**, **d**: coronal section). **(a, b)** Images reconstructed from low-dose raw data using a hybrid iterative reconstruction algorithm (AIDR 3D; Canon Medical Systems, Otawara, Japan). **(c, d)** Images reconstructed from the same raw data using a deep learning reconstruction algorithm (AiCE; Canon Medical Systems). The tumor (asterisks), which appears to have a high density owing to prior transarterial lipiodol marking, is encompassed within the low-density iceball (arrowheads). The images reconstructed using the deep learning reconstruction algorithm provide less image noise and a more conspicuous iceball contour

The application of AI to improve medical image quality has been extensively investigated and is being increasingly implemented in clinical practice. Deep learning reconstruction (DLR) of CT and MRI images is representative and can reduce image noise more effectively than traditional reconstruction methods [16–20]. Tanahashi et al. recently explored the use of DLR in interventional imaging, specifically in CT hepatic arteriography images acquired during TACE for HCC [51]. They quantitatively and qualitatively assessed CT hepatic arteriography images of 27 patients using hybrid-iterative reconstruction and DLR techniques and found that DLR improved the signal-to-noise ratio of small hepatic arteries, contrast-to-noise ratio of tumors, and visualization of tumor-feeding arteries. DLR may also reduce radiation exposure in CT-guided procedures, as it can ensure adequate image quality even with lower radiation doses than those in conventional reconstruction techniques. Matsumoto et al. investigated the radiation dose during CT-guided biopsies and drainage using a 320-detector row CT with DLR and reported that using this system significantly lowered radiation doses compared to conventional CT systems (dose length product: 278 vs. 548 mGy*cm in biopsies and 246 vs. 667 mGy*cm in drainage, both *p* < 0.001) [52]. Although reports on the efficacy of DLR in CT-guided tumor ablation are scarce, dose reduction by DLR may be particularly beneficial in ablation therapies as they generally require higher radiation doses than those in biopsy or drainage [53]. For instance, DLR might be advantageous in CT-guided renal cryoablation, where the radiation dose can be high because of multiple needle insertions and repeated CT scans for iceball monitoring [54–56]. The doses may be reduced with DLR while maintaining the image quality required for implementing the procedure (Fig. 2).

Other deep learning applications for image quality improvement in CT-guided procedures include reduced needle artifacts and the generation of virtual contrast-enhanced images. Cao et al. reported a deep learning model for metal artifact reduction in CT-guided interventional oncology procedures [57]. They scanned CT images with various cryoprobe configurations in a phantom and created images with and without probe artifacts using intensity thresholding. Probes with and without artifacts were segmented and inserted into patient images to simulate procedural images, and a U-net-type model was then trained for metal artifact reduction using these simulated images. When applied to CT images obtained during actual renal cryoablation, this model significantly improved the visual assessment scores by 34–46% for overall image quality, iceball conspicuity, needle tip visualization, target region confidence, and metal artifacts. Pinnock et al. reported a deep learning method using a conditional GAN to generate multi-phase synthetic contrast-enhanced CT images for interventional procedures [58]. They trained the models using pre-procedural CT data from 34 patients undergoing renal cryoablation and demonstrated the feasibility of generating virtual contrast-enhanced CT images of various phases from non-contrast CT. Notably, their model could perform virtual contrast enhancement even on images containing cryoprobes and an iceball that were not present in the training data. Although such a method may have the potential to enable better visualization of target lesions, as in contrast-enhanced CT, without actually administering contrast media during ablation therapies, whether the quality of the synthetic images is sufficiently high and reliable for clinical use remains to be validated.

Additionally, deep learning has the potential to improve the image quality of distal subtraction angiography (DSA) during transcatheter procedures. An inherent limitation of DSA is the presence of misregistration artifacts caused by misalignment between the mask and contrast-enhanced images. To overcome this limitation, some investigators explored the use of deep learning to generate synthetic DSA images without masks, initially focusing on cerebral angiography [59, 60]. Ueda et al. developed a deep learning-based model to generate cerebral DSA-like images using a conditional GAN trained with pairs of dynamic angiograms and DSA without misregistration [59]. The quantitative evaluation showed a sufficiently high coincidence between the DSA-like images generated by the model and the original DSA. Furthermore, a visual evaluation conducted using a test dataset comprising misregistered images demonstrated that the DSA-like images achieved similar or better scores than those by the original DSA. More recently, Crabb et al. reported a similar approach to generate deep learning-based DSA-like images of the hepatic and splenic arteries [61]. This method can potentially address the issue of misregistration artifacts caused by patient motion, respiratory movement of organs, and intestinal peristalsis, which obscure the visualization of target tumors and feeding vessels in transcatheter cancer treatments, such as TACE for HCC. However, further investigations are necessary before its use in clinical practice, including whether deep learning-based DSA ensures sufficient visualization of tumor staining.

## Prediction of treatment outcomes

Predicting treatment outcomes is crucial for selecting appropriate strategies for each patient. Therefore, investigators have pursued AI-based models that provide accurate prognostic predictions after intervention. The development of AI-based predictive models includes multiple steps, such as data extraction, key feature selection, and model construction. The data entered into the model can be clinical, radiological, or both. Clinical data can include patient demographics, laboratory findings, tumor characteristics, and procedure-related data such as ablation parameters. Radiological data can be obtained from radiomics analysis or manual image evaluation. The outcomes predicted from these data include treatment response, survival, or complications. Machine learning can be partially or comprehensively used to construct predictive models [62]. When using clinical or handcrafted radiological features as inputs, machine learning can be employed for feature selection, model building, or both; however, standard statistical methods may also be used for these purposes. In radiomics, machine learning contributes to image processing, feature selection, and final model building. Furthermore, deep learning allows the skipping of multiple steps and direct processing of image inputs to predict outputs [62]. When incorporating clinical and radiomics features into a model, they can be entered simultaneously into models using machine learning. Alternatively, the clinical and radiomics models can be built separately and later combined using methods, such as nomograms, to develop an integrated model.

AI-based predictive models have been frequently reported for the treatment of liver tumors, particularly HCC. Hsieh et al. previously reviewed studies published until 2022 on machine learning and radiomics for the prognosis prediction of TACE and ablation for HCC [62]. In their review, the models for TACE showed an area under the curve (AUC) of 0.81–0.99 in predicting tumor response (responders [complete or partial response] vs. non-responders [stable or progressive disease], mainly based on modified Response Evaluation Criteria in Solid Tumors). The models for ablative therapies showed C-indexes of 0.72–0.73 in predicting progression-free or recurrence-free survivals. In addition, two meta-analyses on the radiomics-based prediction of outcomes after TACE for HCC have been published. The earlier one by Feng et al. included six studies published until October 2022, and showed a pooled sensitivity and specificity of 0.90 and 0.81, respectively, for predicting tumor response [63]. The latter study by Wang et al. included 24 studies published until July 2023 and showed that the radiomics-clinical model achieved C-indexes of 0.88 and 0.80 for predicting treatment response and survival status, respectively [64]. Moreover, Mirza-Aghazadeh-Attari et al. conducted a meta-analysis of studies published until May 2023 to evaluate the radiomics-based prediction of tumor response after radioembolization for liver tumor, showing a pooled sensitivity and specificity of 0.84 and 0.87, respectively [65]. Notably, studies using machine learning techniques to predict the outcomes of liver tumor treatments have been successively published, even after these meta-analyses. The most recent studies published in 2023 or later are summarized in Tables 1 and 2, excluding those included in the aforementioned meta-analyses. In these studies, the models for TACE provided AUC of 0.70–0.96 and 0.80–0.93 for predicting overall survival and tumor response, respectively (Table 1) [66–78]. The models for ablative therapies provided an AUC of 0.83–0.98 for the prediction of local tumor control (Table 2) [79–83].

**Table 1:** Most recent studies using machine learning to predict outcomes of transarterial treatments for hepatocellular carcinoma

Author, Year	Treatment	No. of participants^a^	Outcomes predicted	Input	Methods		Best model performance
					Feature selection	Model construction	
Liu Y 2024 [66]	cTACE	110 (Internal testing by five-fold cross-validation)	TR^b^ (mRECIST)	Clinical data MRI (radiomics)	cML	DL cML Nomogram^c^	AUC: 0.87
Peng G 2024 [67]	cTACE	Training: 248 Test (internal): 107	EHM	Clinical data MRI (radiomics)	cML	cML Nomogram^c^	C-index: 0.83 AUC: 0.83/0.82/0.89/0.95/0.93 for 1/2/3/4/5 yr EHM probability
Wang Q 2024 [68]	TACE with ablation	Training: 172 Test (internal): 75	RFS	Clinical data	cML	Nomogram	C-index: 0.64 AUC: 0.69/0.72/0.75 for 1/3/5 yr RFS
Yang C 2024 [69]	cTACE	Training: 77 Test (internal): 34	OS	Clinical data MRI (radiomics)	cML	cML	C-index: 0.80 AUC: 0.83^d^
Zhang L 2024 [70]	cTACE	Training: 181 Test (external): 186	TR^b^ (mRECIST)	Clinical data CT (handcrafted features)	Standard statistical method	cML	AUC: 0.80
Sun Z 2024 [71]	TACE	Training: 241 Test (internal): 60	OS	Clinical data CT (radiomics)	DL cML	DL cML	C-index: 0.88 AUC 0.96 for 3 yr OS
Chen Y 2024 [72]	TACE	Training: 1,075 Test (internal): 269 Test (external): 414	OS	Clinical data	Standard statistical method	DL	C-index: 0.70 AUC: 0.77/0.73/0.70 for 1/3/5 yr OS
Zhang X 2024 [73]	DEB-TACE	Training: 86 Test (internal): 22	TR^b^ (mRECIST)	Clinical data CT (Radiomics)	cML	cML Nomogram^c^	AUC: 0.93
Liu W 2024 [74)]	TACE HAIC	Training: 1,700 Test (internal): 428 Test (external): 200	OS	Clinical data	cML	cML	AUC: 0.81/0.74/0.70/0.79 for 1/2/3/5 yr OS
İnce O 2023 [75]	cTACE DEB-TACE	188 Training/test (internal) = 7/3	TR^b^ (EASL)	Clinical data MRI (radiomics)	cML	cML	AUC: 0.91
Li J 2023 [76]	DEB-TACE	Training: 201 Test (internal): 87	ALFD	Clinical data	cML	Nomogram	AUC: 0.88
Liang Y 2023 [77]	Postoperative TACE	274 Training/ test (internal) = 8/2	OS RFS	Clinical data	NA	cML	AUC: 0.91/0.94/0.95 for 1/2/3 yr OS, 0.81/0.85/0.83 for 1/2/3 yr RFS
Ma J 2023 [78]	TACE with lenvatinib	Training: 88 Test (internal): 37	TR^b^ (mRECIST)	Clinical data	cML	cML	AUC: 0.91

^a^The definitions of data set terms varied across studies. To avoid ambiguity due to inconsistent terminology, the names of data sets in the table are listed according to the following definitions [84], regardless of the terms used in the original papers: i) Training: a data set used for initial learning to determine model parameters, ii) Validation: a data set used for parameter tuning and model refinement, iii) Test: a data set used to evaluate the final model performance. A test set can be either internal (split from the same pool as the training set) or external (unrelated to the training and internal testing sets, differing from these temporally or geographically)

^b^Responders showed complete or partial response, and non-responders exhibited stable or progressive disease

^c^Nomogram integrated clinical and radiomics models

^d^A mean value of multiple time-dependent AUC values across 6–54 months from enrollment

*TACE* = transarterial chemoembolization, *cTACE* = conventional TACE, *DEB-TACE* = drug eluting beads TACE, *HAIC* = hepatic arterial infusion chemotherapy, *TR* = treatment response, *mRECIST* = modified Response Evaluation Criteria in Solid Tumors, *EASL* = European Association for the Study of the Liver criteria, *EHM* = extrahepatic metastasis, *RFS* = recurrence free survival, *OS* = overall survival, *ALFD* = acute liver function deterioration, *MRI* = magnetic resonance imaging, *CT* = computed tomography, *cML* = conventional machine learning, *DL* = deep learning, *NA* = not applicable, *AUC* = area under the curve

**Table 2:** Most recent studies using machine learning to predict outcomes of ablation therapies for liver tumors

Author, Year	Treatment	No. of participants^a^	Outcomes predicted	Input	Methods		Best model performance
					Feature selection	Model construction	
Hamed AA 2024 [79]	RFA for HCC	111 Training/Test (internal) = 7/3	RFS	Clinical data	NA	cML	AUC: 0.80 for 1 yr RFS
Sato M 2023 [80]	RFA for HCC	Training: 1,422 Validation: 178 Test (internal): 178	OS	Clinical data	NA	DL	C-index: 0.69
Ren H 2023 [81]	MWA for HCC	Training: 607 Test (external): 299	LTP	Clinical data	cML	cML	AUC: 0.90 for LTP within 2 yrs
Shahveranova A 2023 [82]	MWA for CRLM	42^b^	LTP	Clinical data MRI (radiomics)	cML	cML	AUC: 0.98 for LTP within 6 months
Tabari A 2023 [83]	RFA or MWA for HCC^c^	97 Training/validation/ test (internal) = 6/2/2	Pathological response^d^	Clinical data MRI (radiomics)	cML	cML	AUC: 0.83

^a^The definitions of data set terms varied across studies. To avoid ambiguity due to inconsistent terminology, the names of data sets in the table are listed according to the following definitions [84], regardless of the terms used in the original papers: i) Training: a data set used for initial learning to determine model parameters, ii) Validation: a data set used for parameter tuning and model refinement, iii) Test: a data set used to evaluate the final model performance. A test set can be either internal (split from the same pool as the training set) or external (unrelated to the training and internal testing sets, differing from these temporally or geographically)

^b^No internal or external testing was performed

^c^RFA or MWA were performed as bridge to liver transplant

^d^Histopathology was assessed at the time of liver transplant

*RFA* = radiofrequency ablation, *MWA* = microwave ablation, *HCC* = hepatocellular carcinoma, *CRLM* = colorectal carcinoma liver metastases, *RFS* = recurrence free survival, *OS* = overall survival, *LTP* = local tumor progression, *MRI* = magnetic resonance imaging, *NA* = not applicable, *cML* = conventional machine learning, *DL* = deep learning, *AUC* = area under the curve

While most studies on AI-based outcome prediction thus far have been conducted on liver tumors, a few reports have shown similar results for lung tumor ablation [85–87]. Crombé et al. investigated a radiomics model to predict local tumor progression (LTP) following RFA of colorectal cancer lung metastases [85]. Conventional machine learning algorithms were trained using radiomic features extracted from the ablation zone segmented on early follow-up CT, and the best model showed a moderate AUC of 0.72. They suggested that the performance of their radiomics model might have been limited by the capture of inflammation, intra-alveolar hemorrhage, cavitation, and fistulization during complicated procedures.

As described above, AI-based predictive models have demonstrated moderate-to-high predictive performance. Such AI-based prognostication may be useful for supporting treatment decision-making [88–90]. However, the study results should be interpreted with caution in terms of reproducibility, given the diversity of the proposed models. The details of the method vary widely among studies regarding input features (clinical, radiomics, or both), imaging modality, image processing method, and machine learning algorithms [62–65]. Furthermore, the performance of these models has not always been evaluated using external test cohorts. Hence, the superiority of any particular algorithm is not evident and requires further investigation.

## Detection of post-treatment recurrence

AI-based techniques for lesion detection in radiological images have been studied extensively. For example, there are a number of reports on the AI-based detection of pulmonsary nodules on CT [91], and such AI models have been clinically implemented. Consequently, AI is expected to be useful in detecting recurrent lesions after image-guided therapies. Early detection of local recurrences on follow-up images is important to promptly consider a secondary strategy, including reintervention. However, detecting local recurrence on follow-up images can be more complicated than detecting de novo lesions because of post-treatment changes in the region of interest. In image-guided tumor ablation, LTP is identified as a nodular enhanced focus within or adjacent to the ablation zone [92, 93]. To detect early LTP, a small focus needs to be extracted from the treatment area, where radiological changes due to reactive inflammation and scarring are usually observed. Despite this difficulty, some investigators have used AI to facilitate LTP detection on follow-up imaging. Yin et al. investigated the efficacy of machine learning-based radiomics analysis for detecting LTP on follow-up contrast-enhanced CT after thermal ablation of HCC and metastatic liver tumors [94]. Radiomics features were extracted from the region of interest, including the ablation zone and surrounding liver parenchyma on follow-up CT images, and models were trained using the selected features. The best-performing model achieved an accuracy of 92.7% and an AUC of 0.97 for detecting LTP. Lim et al. developed a deep learning method to detect LTP after RFA or MWA for HCC using follow-up CT images [95]. Their deep CNN model used 3D patches extracted from arterial-phase CT images to detect LTP. The model performance on test datasets demonstrated an accuracy of 97.6% and an AUC of 0.99 in detecting LTP.

## Current issues and future directions

Research has explored a wide variety of AI models for various tasks in interventional oncology procedures. As AI technology advances, more AI-based methods will be developed. Similar to AI, extended reality (virtual, augmented, and mixed reality) and robotics have gained attention as cutting-edge technologies that can be useful in interventional oncology [26, 96]. The integration of AI with these technologies may further enhance advanced image-guided cancer treatment [97]. The potential benefits of introducing AI include not only improved workflow and treatment outcomes, but also a reduction in radiation exposure to patients and physicians—an inherent issue in image-guided interventions. Although its significance in interventional oncology procedures remains to be validated, the evolution of X-ray fluoroscopy and DSA technologies by AI-based image processing may contribute to greatly reduced intraoperative radiation doses [98, 99].

However, most AI-based methods discussed in this review are still in the research phase, and few have been implemented in clinical practice. Investigators have utilized various algorithms to develop and test AI models, making objective evaluation and impartial comparison of model performance across studies difficult, even among those aiming for similar tasks. Therefore, the real-world performance and clinical reliability of AI-based methods must be interpreted carefully. Additionally, the relatively small datasets available in the field of interventional radiology compared with those in diagnostic radiology could be a limitation in the development of AI models [24]. The establishment and widespread clinical use of highly reliable AI models across various areas and institutions are still uncertain. The Cardiovascular and Interventional Radiological Society of Europe outlines several conditions for the widespread use of AI in daily clinical practice, including ensuring sufficient accuracy and reliability, seamless integration with procedural workflows, and meeting regulatory requirements [100]. They also highlighted the need to integrate computer science and AI knowledge into education and training because it might become as important for interventional radiologists as knowledge in biostatistics. Moreover, when employing AI-based technologies, we need to recognize fairness issues in AI, which are caused by potential biases from data, algorithms, and AI clinician/patient interactions [101].

In conclusion, AI has the potential to enhance various aspects of interventional oncology practice, from treatment planning to post-treatment follow-up. For AI technologies to be widely adopted in interventional oncology procedures, further investigations of their reliability and clinical utility are necessary. Despite this challenge, various AI technologies will be incorporated into interventional oncology in the near future, because of the rapid research progress in this field.
